# Multidimensional Impulsivity Profile in Young Adults Aged 16 to 25 with Borderline Personality Disorder: A Study Based on the UPPS-P Model

**DOI:** 10.3390/jcm14197109

**Published:** 2025-10-09

**Authors:** Anaïs Mungo, Marie Delhaye, Matthieu Hein

**Affiliations:** 1 Department of Psychiatry, CH Le Domaine-ULB, Université Libre de Bruxelles (ULB), 1420 Braine l’Alleud, Belgium; 2 Unit 2, Department of Psychiatry, La Ramée, Epsylon, 1180 Brussels, Belgium; marie.delhaye@umons.ac.be; 3 Laboratoire de Psychologie Médicale et Addictologie (ULB312), CHU Brugmann, Université Libre de Bruxelles (ULB), 1020 Brussels, Belgium

**Keywords:** borderline personality disorder, impulsivity, negative urgency, UPPS-P model, young adults, early intervention, emotion dysregulation

## Abstract

**Background**: Borderline Personality Disorder (BPD) often emerges during adolescence and young adulthood, a period marked by heightened vulnerability to impulsivity and affective dysregulation. While impulsivity is a core feature of BPD, its multidimensional expression in this age group remains insufficiently documented. This study examined impulsivity traits in young adults with BPD, their associations with depressive and anxiety symptoms, and their links to risk behaviors. **Methods**: A total of 160 participants aged 16–25 were recruited in Belgium between 2021 and 2023: 44 with BPD from inpatient and outpatient psychiatric services and 116 healthy controls from schools and universities. Assessments included the short UPPS-P, Beck Depression Inventory-II (BDI-II), State-Trait Anxiety Inventory (STAI-T), and the Diagnostic Interview for Borderlines–Revised (DIB-R). Logistic regressions with robust errors and Kendall’s tau-b correlations were used. **Results**: Compared with controls, individuals with BPD scored higher on all UPPS-*p* subscales except Sensation Seeking (e.g., Negative Urgency: 14 vs. 10, *p* < 0.001). Logistic regression identified Negative Urgency (OR = 5.31, 95% CI: 2.07–13.62, *p* = 0.001) and Positive Urgency (OR = 3.26, 95% CI: 1.37–7.75, *p* = 0.007) as independent predictors of BPD. Within the BPD group, depressive and anxiety symptoms correlated with several UPPS-P dimensions, notably Negative Urgency and Lack of Perseverance. Suicide attempts were associated with the DIB-R total score, BDI-II, and STAI-T, while substance use was linked to the DIB-R impulsivity subscale and STAI-T. **Conclusions**: Emotional impulsivity—particularly Negative Urgency—emerges as a central feature of BPD in emerging adulthood. Its interplay with depressive and anxiety symptoms, and its associations with suicidal and addictive behaviors, support a dual-level conceptualization of impulsivity as both a dispositional trait and a state-dependent clinical risk factor.

## 1. Introduction

### 1.1. Borderline Personality Disorder (BPD): Definition and Clinical Complexity

Borderline Personality Disorder (BPD) is a severe psychiatric disorder that typically emerges during adolescence or early adulthood, a critical developmental window during which dysfunctional personality traits may consolidate into a stable clinical pattern [1,2]. BPD is characterized by pronounced emotional instability, unstable interpersonal relationships, disturbances in self-image, and recurrent impulsive and self-harming behaviors [3].

These symptoms often manifest as impaired emotion regulation, a chronic sense of emptiness, hypersensitivity to rejection, and frequent engagement in risky behaviors to alleviate psychological distress [1]. Such behaviors may include self-injury, suicide attempts, substance use (10.6% to 14.6%) [4], eating disorders (16.9% to 53.8%) [5], and unprotected sexual activity (21%) [5]. Far from being more expressions of impulsivity, these behaviors frequently serve as maladaptive strategies for emotion regulation in response to intense negative affect [6] and tend to escalate during acute episodes of psychological distress, especially among individuals with pronounced borderline traits [7].

### 1.2. Impulsivity in BPD: Diagnostic Status and Explanatory Models

Impulsivity is thus a cardinal symptom of BPD, included in the diagnostic criteria of international classification systems such as the DSM-5, ICD-10, and ICD-11 [3,8,9]. It is commonly defined as a persistent tendency to act without forethought, often in high-risk domains such as excessive spending, risky sexual behavior, or substance use [1]. The alternative model for personality disorders in Section III of the DSM-5 reinforces the centrality of impulsivity, designating it as a core pathological trait of personality functioning [3].

Theoretical models of BPD also place impulsivity at the heart of the disorder. Linehan’s biosocial model conceptualizes it as a dysfunctional behavioral response to severe emotional dysregulation, shaped by a chronically invalidating environment [10]. From a psychodynamic perspective, Kernberg interprets impulsivity in BPD as a behavioral manifestation of unintegrated primitive aggression. This aggression may be directed outward—through acting-out behaviors or risk-taking—or inward, in the form of self-injury or self-destructive behaviors. It originates from split internal object representations oscillating between idealized and persecutory poles, which are typical of borderline personality organization. The inability to integrate these contradictory representations, combined with the predominant use of primitive defense mechanisms such as splitting, projective identification, or devaluation, hinders the development of a structured superego and impairs affect regulation, thereby predisposing the individual to impulsive acting out [11].

Empirical data confirm the high prevalence of impulsivity among individuals with BPD, who score significantly higher than healthy controls or other clinical groups on self-report measures such as the Barratt Impulsiveness Scale (BIS-11), with the exception of comorbid cases involving Attention-Deficit/Hyperactivity Disorder (ADHD), in which impulsivity levels are similarly elevated [12]. Clinically, impulsivity is not only a core symptom but also a predictor of severity, linked to: increased suicide risk, relapse, affective and interpersonal instability and poorer prognosis. This underscores the therapeutic importance of targeting impulsivity [13,14,15].

While prior studies have identified elevated impulsivity in BPD, few have systematically explored how distinct impulsivity dimensions, as measured by the UPPS-P model, relate to clinical severity and comorbid symptomatology in young adults—a gap the current study aims to address.

### 1.3. Multidimensional Impulsivity: Neurocognitive and Trait-Based Perspectives

Impulsivity is a core construct in psychopathology, implicated in a wide range of psychiatric disorders such as BPD and ADHD, Cluster B personality disorders (a group of disorders in the DSM-5, characterized by emotional, dramatic, or erratic behavior, including Antisocial, Histrionic, Narcissistic, and Borderline Personality Disorders), substance use disorders, and bipolar disorder [14]. However, it should not be viewed as a homogeneous phenomenon. Despite its frequent appearance in nosological classifications, impulsivity is a complex, multifaceted construct that defies simple definition [16].

According to Moeller et al. [14], impulsivity may be defined as “a predisposition toward rapid, unplanned reactions to internal or external stimuli without regard to the negative consequences of these reactions.” This definition highlights a failure of inhibitory control, often associated with risk-taking behaviors.

Psychologically, several dimensions of impulsivity have been identified. Barratt’s model distinguishes three core components: motor impulsivity (acting without thinking), cognitive impulsivity (making rapid decisions without thorough analysis), and lack of planning (difficulty anticipating consequences) [14]. These dimensions are incorporated into tools like the BIS-11, widely used in clinical research, including in studies of BPD [14].

Neuropsychologically, impulsivity is frequently conceptualized as an impairment in inhibitory control and broader executive functions supported by prefrontal structures. However, some findings suggest a more nuanced understanding [17]. For instance, Sebastian et al. [17] demonstrated that in BPD, impulsivity does not necessarily reflect a baseline inhibitory deficit, but rather a secondary response to emotional dysregulation, particularly in the presence of intense negative emotions [17]. Several studies have shown that impulsivity in BPD is strongly modulated by emotional state, especially when negative affect is salient and tied to personal trauma or conflict [18,19,20].

Neuroimaging data further underscore this complexity. Franczak et al. [21] reported that impulsivity in BPD is associated with alterations in prefrontal and limbic regions, whereas impulsivity in ADHD is more strongly linked to abnormalities in the striatum and caudate nucleus. These neurobiological distinctions support the notion that while impulsivity may be a shared symptom across disorders, its underlying mechanisms differ, with important implications for differential diagnosis and treatment planning [21].

Impulsivity can also be examined from a dimensional perspective, notably through the UPPS-P model proposed by Whiteside and Lynam and later expanded by Cyders and Smith [22,23]. This model has greatly advanced the understanding of impulsivity by distinguishing five dimensions: negative urgency (tendency to act rashly under intense negative emotions), positive urgency (impulsivity triggered by intense positive emotions), lack of premeditation (difficulty anticipating consequences), lack of perseverance (inability to sustain effort on demanding or boring tasks), and sensation seeking (propensity to pursue novel or risky experiences). This multidimensional framework enables more precise profiling of impulsive tendencies across disorders by identifying specific emotional triggers for each type of impulsivity [22,23].

For example, negative urgency has been associated with self-destructive behaviors such as self-injury and disordered eating, whereas sensation seeking is more commonly linked to substance use and risk-taking [24,25]. Lack of premeditation, meanwhile, is implicated in antisocial and delinquent behaviors [23].

Studies using the UPPS-P model have shown that these dimensions are differentially associated with a range of mental health conditions, including BPD, substance use disorders, mood disorders, and ADHD [24,26]. The model offers a more refined means of predicting problematic behaviors and tailoring therapeutic interventions to specific impulsivity profiles. Moreover, it has been validated across multiple languages and age groups, including adolescents and young adults, reinforcing its clinical utility [24,25].

Thus, far from being a simple tendency to “act without thinking,” impulsivity emerges as a multidimensional construct with neurocognitive, affective, and behavioral underpinnings that merit thorough investigation, particularly in complex disorders such as BPD [15].

Given the model’s empirical relevance, the UPPS-P framework will serve as the theoretical and methodological foundation of the present study, which aims to examine specific impulsivity profiles in BPD.

### 1.4. Emotional Impulsivity in BPD: Empirical Evidence, Modulators, and Clinical Relevance

Impulsivity is a major hallmark of BPD and is embedded in its diagnostic criteria. Numerous studies have confirmed that individuals with BPD consistently show significantly higher levels of impulsivity than the general population. This is particularly evident in elevated scores on standardized measures such as the Barratt Impulsiveness Scale (BIS-11) and the UPPS-P Impulsive Behavior Scale [17,19,27], reinforcing the idea that impulsivity—especially behavioral disinhibition—is a core psychopathological feature of BPD.

Recent findings, particularly those presented by Waite et al. [5], provide key insights into the emotional mechanisms underlying impulsivity in BPD. The following sections synthesizes these data within a broader clinical perspective.

Self-report assessments, particularly the UPPS-P questionnaire, consistently indicate heightened impulsivity among individuals with BPD, compared not only to healthy controls but also to other clinical populations such as those with depressive disorders [19,28,29,30]. These findings underscore the salience of impulsivity in BPD clinical profiles.

However, results from behavioral tasks offer a more nuanced picture. A meta-analysis revealed a weak correlation between self-reports and behavioral measures of impulsivity, suggesting that these methods tap into different, yet complementary, facets of the construct [31,32]. For instance, although women with BPD often report high impulsivity on self-assessment scales, they do not necessarily perform worse on experimental inhibition tasks such as the Stroop Test, Stop Signal Task, or Antisaccade Task [19].

This dissociation is also evident across impulsivity subtypes. Negative urgency—defined as the tendency to act impulsively under conditions of emotional distress—closely overlaps with the concept of emotional dysregulation [33]. It can be assessed via distress tolerance tasks, such as the Paced Auditory Serial Addition Task (PASAT) [34], where premature termination of the task reflects heightened sensitivity to emotional discomfort. Some studies have found that individuals with BPD tend to quit such tasks more quickly, indicating increased affective vulnerability [35,36], though these findings are not always replicated [37].

Sensation seeking, although frequently self-reported as elevated in BPD patients, does not consistently translate into riskier behaviors in laboratory settings. While some studies observe increased risk-taking behaviors [38,39], others find no significant differences compared to control groups [28], suggesting that sensation-seeking behavior may be modulated by contextual or emotional variables.

The lack of perseverance remains relatively underexplored through behavioral measures. Although some studies have identified correlations between this trait and performance on inhibition tasks [40], empirical data remain limited and often contradictory [19,41]. Certain findings suggest that individuals with BPD demonstrate lower distress tolerance in demanding or repetitive tasks, which may reflect a tendency toward premature task abandonment [35].

As for lack of premeditation, this dimension is frequently assessed using delay discounting paradigms, which require participants to choose between smaller immediate rewards and larger delayed rewards. Individuals with BPD tend to prefer immediate gratification, indicating difficulty anticipating long-term consequences of their actions [42,43]. However, this tendency may be exacerbated by comorbid conditions, particularly substance use disorders [43].

However, not all impulsivity-related traits show consistent associations with BPD symptomatology. In particular, findings regarding cognitive dimensions of impulsivity—such as lack of premeditation, lack of perseverance, and sensation seeking—are often mixed or null. Several studies, especially those conducted in nonclinical or subclinical populations, have reported no significant relationships between these traits and borderline features. These inconsistencies suggest that emotionally driven facets, such as negative and positive urgency, may be more central to the disorder, while “cold” impulsivity traits may reflect more general or transdiagnostic tendencies. This perspective is further supported by recent work demonstrating an absence of significant associations between executive functions and BPD symptomatology in nonclinical samples (e.g., Veerapandian et al., 2023), underscoring the need for a nuanced, multidimensional understanding of impulsivity in BPD [44].

Recent data reveal a particularly pronounced impulsivity profile in BPD, especially regarding negative urgency, while behavioral task results remain heterogeneous. These discrepancies highlight the importance of viewing impulsivity as a multidimensional phenomenon influenced by emotional state, context, and the evaluation method employed.

These observed differences in impulsivity subtypes—particularly negative urgency and lack of premeditation—support the relevance of examining UPPS-P dimensions in clinical populations, a focus taken up in the present research

Moreover, several methodological limitations persist in the literature. Few studies have focused specifically on youth, even though adolescence and emerging adulthood (ages 16–25) represent a critical window for the emergence of borderline traits. This is precisely the period when impulsivity tends to be most intense and may decisively influence psychosocial development [45].

While certain studies have demonstrated robust associations between negative urgency and BPD symptoms, comparative or longitudinal clinical investigations remain scarce. Many studies suffer from small sample sizes or limited methodologies, which hampers generalizability [5,23,46].

Furthermore, most studies compare BPD patients with healthy controls, rarely including other psychiatric groups. This limits the differential value of the findings. For example, the study by Linhartová et al. (2019), which compared BPD patients to individuals with ADHD, revealed distinct impulsivity profiles: while impulsivity in ADHD is primarily motor-based, BPD-related impulsivity appears more context-dependent and emotionally modulated, particularly in distressing situations [27].

Recent findings, including those by Waite et al. [5], confirm this distinction. They show that negative emotional dysregulation is closely associated with negative urgency, lack of premeditation, and perseverance deficits. In contrast, positive emotion dysregulation seems linked to sensation seeking [5]. These results enhance the understanding of BPD by suggesting that impulsivity in this disorder does not merely stem from impaired control but rather unfolds within a broader emotional dynamic [5].

Building on this affective modulation framework, the current study will further investigate the extent to which emotional impulsivity, especially negative urgency, predicts BPD status beyond general affective symptoms.

Finally, it is essential to consider the role of major comorbidities such as depression, anxiety, and substance use, which are frequently associated with BPD and significantly impact impulsivity levels [5,19]. Sebastian et al. have highlighted the potential influence of uncontrolled comorbidities—particularly ADHD—on the interpretation of impulsivity in BPD, emphasizing the need for rigorous control of these variables in future research [17].

Beyond internalizing disorders, externalizing comorbidities such as substance use disorders and antisocial behaviors are also frequently associated with BPD. These conditions not only share impulsivity as a core feature but may exacerbate emotional and behavioral dysregulation. Importantly, traits like sensation seeking and lack of premeditation—often elevated in such comorbidities—can compound the clinical picture and challenge diagnostic clarity [13,23,24,25].

To disentangle these overlapping influences, the present study examines how affective and behavioral variables commonly associated with multiple psychiatric disorders—such as depressive symptoms, anxiety, and substance use—modulate impulsivity patterns specifically within the BPD population.

While these variables are described in the literature as transdiagnostic risk factors, our data do not allow us to explore their expression across different diagnostic categories. Clinically, impulsivity constitutes a transversal risk factor in BPD. It is associated with increased suicidal behavior, relapse, risk-taking conduct, and affective and interpersonal instability. It also predicts disorder severity and long-term prognosis, making it a key therapeutic target [14,15]. Psychotherapeutic interventions such as Dialectical Behavior Therapy (DBT) explicitly aim to improve emotion regulation and thereby reduce impulsive behaviors [1,15].

In summary, deepening our understanding of impulsivity in BPD—by relying on dimensional models like the UPPS-P, integrating developmental and comorbidity factors, and employing diverse methodologies—represents a major clinical and theoretical challenge. It could foster more accurate diagnostic differentiation, better tailored interventions, and improved outcome prediction in affected young adults.

### 1.5. Objectives

This study aims to better characterize the multidimensional impulsivity profile of young adults with Borderline Personality Disorder (BPD), using the UPPS-P model as a theoretical and measurement framework. Drawing on current empirical findings and theoretical models of BPD and impulsivity, we formulated the following hypotheses:

First, we hypothesize that individuals with BPD will display significantly higher scores on specific UPPS-P subscales—namely, Negative Urgency, Positive Urgency, and Lack of Premeditation—compared to healthy controls. This profile is expected to reflect both heightened emotional impulsivity and reduced anticipatory control.

Second, we postulate that Negative Urgency will be a significant predictor of BPD group membership, independent of affective symptoms. This will be tested through multivariate logistic regression analyses.

Third, we anticipate that impulsivity traits measured by the UPPS-P will be primarily associated with the impulsive dimension of the Diagnostic Interview for Borderlines—Revised (DIB-R), reflecting the overlap between dispositional impulsivity and clinical behavioral dysregulation. Exploratory correlations will also be examined with the affective, cognitive, and interpersonal dimensions of the DIB-R.

Finally, we will examine whether specific clinical indicators—namely suicidal behaviors and substance use (alcohol, tobacco, illicit drugs)—are associated with the severity of borderline symptoms (DIB-R), as well as with trait anxiety (STAI-T) and depressive symptoms (BDI-II). These variables are well-established markers of psychological distress and may help clarify the broader clinical context in which impulsivity manifests.

## 2. Materials and Methods

### 2.1. Population

The study included both clinical and non-clinical populations. Participants in the clinical group were recruited between January 2021 and October 2023 from inpatient and outpatient services of the Adolescent and Adult Psychiatry Departments of an academic general hospital located in the Brussels metropolitan area. Inclusion criteria for the clinical group were a diagnosis of Borderline Personality Disorder (BPD) based on DSM-5 criteria, confirmed by the treating psychiatrist and by an experienced clinical investigator using the semi-structured Diagnostic Interview for Borderlines—Revised (DIB-R), an age between 16 and 25 years, and sufficient proficiency in spoken and written French. Exclusion criteria were refusal to participate in the study, inability to complete the assessments, presence of an intellectual disability, current or past diagnosis of a psychotic disorder (e.g., schizophrenia, autism spectrum disorder), or severe somatic illness (e.g., cancer, cardiac or renal failure, progressive neurological disease) likely to compromise short-term prognosis.

Importantly, participants with BPD were recruited from both inpatient and outpatient services, regardless of current symptom severity or treatment phase. Inclusion was determined solely by the ability to complete self-report questionnaires and the presence of a confirmed BPD diagnosis, with no exclusion based on psychiatric comorbidities or acute clinical states. Consequently, the clinical sample reflects the heterogeneity and fluctuating course of BPD presentations typically encountered in real-world psychiatric settings.

Participants in the non-clinical group were recruited between 2021 and 2023 from secondary schools in Wallonia, northern Brussels, and southern Brussels, in order to obtain a representative sample, as well as from the Faculty of Medicine and other faculties of the Université Libre de Bruxelles (ULB). Additional recruitment was carried out through posters displayed in the university hospital. Inclusion criteria for the control group were an age between 16 and 25 years and fluency in French. Exclusion criteria were refusal to participate in the study, inability to complete the assessments, current or past psychiatric disorder or family history of psychiatric disorders, and severe somatic illness as defined above.

**Figure 1:**
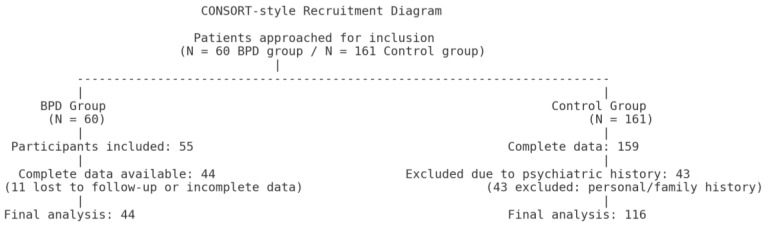
Consort Diagram.

For the borderline personality disorder (BPD) group, 60 patients were initially approached. Among them, 55 were included in the study, but only 44 had complete datasets available for analysis due to loss to follow-up or incomplete data (Figure 1).

For the control group, 161 participants were initially included. Complete data were available for 159 participants. After excluding those with personal or family psychiatric history, 116 participants were retained for the final analysis (Figure 1).

### 2.2. Method

All participants were provided with a detailed explanation of the study procedures, and written informed consent was obtained prior to enrollment. For participants under the age of 18, the written consent of a parent or legal guardian was required. All questionnaires were administered in French.

Only individuals meeting the DSM-5 diagnostic criteria for Borderline Personality Disorder were included in the clinical group. The diagnosis was confirmed through a prior assessment using the Diagnostic Interview for Borderlines—Revised (DIB-R).

The DIB-R is a semi-structured interview that provides both quantitative and qualitative evaluation of the core dimensions of BPD: affect, cognition, impulsive behavior, and interpersonal functioning. Each item is scored on a scale from 0 (absent) to 2 (clearly present), with an intermediate score of 1 (probable). Items are grouped into summary statements and converted into standardized dimension scores according to a detailed scoring procedure. The total DIB-R score ranges from 0 to 10. The DIB-R has demonstrated excellent psychometric properties, including baseline test–retest reliability, interrater reliability, and longitudinal stability of borderline symptoms, with kappa coefficients exceeding 0.75 across all domains [47].

Depressive symptoms were assessed using the Beck Depression Inventory–II (BDI-II) [48]. This self-report questionnaire consists of 21 items, each rated on a 4-point Likert scale ranging from 0 (absence of symptom) to 3 (severe symptom expression). The total score ranges from 0 to 63, with higher scores indicating greater severity of depressive symptoms. The BDI-II evaluates a broad range of cognitive, affective, and somatic symptoms associated with depression, such as sadness, pessimism, self-criticism, fatigue, and changes in appetite or sleep. It is widely used in both clinical and research settings due to its robust psychometric properties and has demonstrated excellent internal consistency and validity across diverse populations. For interpretative purposes, total scores can be categorized as follows: 0–13 indicates minimal or no depression, 14–19 corresponds to mild depression, 20–28 to moderate depression, and 29–63 to severe depression [48].

Trait anxiety was assessed using the Trait subscale of the State-Trait Anxiety Inventory (STAI-T), developed by Spielberger, Gorsuch, and Lushene [49]. The STAI is a validated self-report questionnaire composed of two distinct 20-item subscales: the State Anxiety Scale (STAI-S), which measures transitory emotional states, and the Trait Anxiety Scale (STAI-T), which evaluates stable, enduring tendencies to experience anxiety across time and situations. In the present study, only the Trait Anxiety subscale (STAI-T) was administered, focusing on participants’ general predisposition to anxiety. Each item is rated on a 4-point Likert scale, with higher scores indicating greater trait anxiety. The STAI-T is widely used in clinical and research settings and has demonstrated strong psychometric properties, including high internal consistency and good construct validity. While the STAI does not provide universally standardized diagnostic cut-off scores, previous literature suggests that scores ≥ 45 may reflect elevated anxiety levels, whereas scores between 38 and 44 may be considered moderate, and scores ≤ 37 as low. However, these thresholds should be interpreted cautiously and always in relation to the population norms and clinical context [49].

Impulsivity was assessed using the short French version of the UPPS-P Impulsive Behavior Scale [24]. This self-report questionnaire comprises 20 items that evaluate five distinct dimensions of impulsivity, with four items per subscale:

- Negative Urgency (tendency to act rashly under negative emotions),
- Positive Urgency (tendency to act rashly under positive emotions),
- Lack of Premeditation (acting without thinking ahead),
- Lack of Perseverance (difficulty staying focused on tasks), and
- Sensation Seeking (preference for novel and exciting experiences).

Each item is rated on a 4-point Likert scale, ranging from 1 (“strongly agree”) to 4 (“strongly disagree”), with higher scores indicating higher impulsivity levels.

The scale has demonstrated satisfactory internal consistency in French-speaking populations, with Cronbach’s alpha coefficients reported as follows:

Negative Urgency (α = 0.78), Positive Urgency (α = 0.70), Lack of Premeditation (α = 0.79), Lack of Perseverance (α = 0.84), and Sensation Seeking (α = 0.83) the same paper also reports Cronbach’s α for the long French version of the UPPS-P at approximately 0.77–0.83, and high test–retest reliability for the short form (r = 0.84–0.92) [24].

We also collected self-reported sociodemographic and clinical data, including information on gender, age, and educational level, as well as student status. Participants were asked to report any history of somatic illness or surgical interventions, and to indicate the presence of psychiatric comorbidities, psychotropic medication, and ongoing psychological follow-up.

Additional variables included tobacco use, alcohol consumption, and illicit drug use, along with a personal history of suicide attempts and the number of such attempts. Finally, we assessed the presence of a family history of psychiatric disorders.

### 2.3. Statistical Analyses

All statistical analyses were conducted using Stata software version 14. Participants were categorized into two groups: a control group of normative individuals and a clinical group composed of individuals diagnosed with BPD.

Categorical variables were described using frequencies and percentages, while continuous variables were summarized using medians and interquartile ranges (P25–P75). Between-group comparisons were performed using the Wilcoxon–Mann–Whitney test for continuous variables and the Chi-square test or Fisher’s exact test for categorical variables, depending on expected cell counts.

Group differences in short UPPS-P subscale scores were further examined using quantile regression analyses to determine whether these differences remained significant after adjustment for potential confounding factors. These included: gender, psychiatric comorbidities, smoking, illicit drug use, family history of psychiatric disorders, number of past suicide attempts, current psychological follow-up, psychotropic medication, Beck Depression Inventory (BDI) score, and Trait anxiety score from the STAI-T.

In addition, a multivariate logistic regression analysis was performed to examine whether specific short UPPS-P traits—particularly Negative Urgency and Positive Urgency—significantly predicted BPD group membership, while controlling for gender, BDI score, and STAI-Trait score. In order to take into account the presence of potential heteroscedasticity, the robust standard errors method was used to allow a more reliable estimation of ORs and *p*-values obtained during these different logistic regression analyses. Finally, the validity of the final logistic regression model (adequacy and specificity) was checked using the Hosmer and Lemeshow test and the Link test.

Following the conditions of use of multivariate logistic regression analyses (number of subjects per cofactor ≥ 10), each of the two groups of patients for this study had to contain at least 40 subjects (10 subjects * 4 cofactors) to ensure the validity of the analyses performed, which was achieved in this study.

To further explore associations across the entire sample, tau-b Kendall correlation analyses were conducted between short UPPS-P subscale scores and several clinical variables, including total and subdimension scores from the DIB-R, BDI scores, STAI-Trait scores, substance use, and the number of suicide attempts.

Statistical significance was set at *p* < 0.05. A Bonferroni correction was applied to adjust for multiple testing where applicable.

### 2.4. Ethics Statement

This research protocol was approved by the Ethics Committee of the Erasme-ULB Hospital Faculty, Brussels, Belgium (reference: P2020/111). Written informed consent to participate in the study was obtained from all participants aged 18 years or older, and from their legal guardians or next of kin for those under the age of 18.

## 3. Results

### 3.1. Sample Description

The sample consisted of 160 participants aged 16 to 25 years, divided into a clinical group of 44 young adults diagnosed with BPD and a control group of 116 participants with no psychiatric history. There were no significant differences between the groups in terms of age (median age: 17 years for the BPD group vs. 18 years for controls, *p* = 0.051) or educational level (*p* = 0.237). However, the BPD group had a significantly higher proportion of females (95.4% vs. 72.4%, *p* = 0.001) (Table 1).

Psychiatric comorbidities, outpatient psychological follow-up, and psychotropic medication use were exclusively observed in the BPD group (*p* < 0.001). This group also reported significantly higher rates of smoking (36.4% vs. 9.5%, *p* < 0.001), illicit substance use (47.7% vs. 0%, *p* < 0.001), and a family history of psychiatric disorders (88.6% vs. 0%, *p* < 0.001). Past suicide attempts were reported by 77.3% of BPD patients, compared to none in the control group (*p* < 0.001) (Table 1). The distribution of specific comorbid diagnoses within this group is presented in Appendix A Table A1.

On a psychometric level, individuals with BPD scored significantly higher than controls across all short UPPS-P subscales, except for Sensation Seeking. The most discriminative median scores were as follows: Negative Urgency (14 vs. 10, *p* < 0.001), Positive Urgency (14 vs. 12, *p* < 0.001), Lack of Premeditation (11 vs. 8, *p* < 0.001), and Lack of Perseverance (11 vs. 7, *p* < 0.001). No significant difference was found for Sensation Seeking (*p* = 0.126) (Table 2).

Depressive symptoms (BDI-II) and trait anxiety (STAI-T) scores were also significantly higher in the BPD group (33 vs. 12, *p* < 0.001; and 66 vs. 45, *p* < 0.001, respectively) (Table 3).

The severity of borderline symptomatology within the clinical group was assessed using the Diagnostic Interview for Borderlines—Revised (DIB-R). Median scores across the four subdimensions—affective, cognitive, impulsive, and interpersonal—as well as the total score, are presented in Table 4.

### 3.2. Quantile Regression (Table 5)

Group differences in impulsivity traits were examined using adjusted quantile regression models on short UPPS-P subscale scores. These models assessed the extent to which BPD group membership was associated with elevated impulsivity levels, controlling for the following confounding factors: gender, psychiatric comorbidities, smoking, substance use, family psychiatric history, number of suicide attempts, psychological follow-up, psychotropic treatment, BDI-II score, and STAI-Trait score.

Results are presented in Table 2. They indicate that Negative Urgency remained significantly associated with BPD, with an adjusted coefficient of 9.9 (standard error = 3.7), suggesting a substantial increase in this trait among BPD patients, independent of confounding factors. Positive Urgency also remained significantly elevated, with an adjusted coefficient of 4.8 (SE = 2.4). In contrast, Lack of Premeditation and Lack of Perseverance, although significant in univariate analysis, did not remain significant after adjustment. These quantile regression analyses confirm that emotional urgency traits—particularly Negative Urgency—are robust differentiating markers of BPD in young adults, beyond potential psychological and psychopathological confounders.

### 3.3. Multivariate Analysis: Predictors of BPD Group

A multivariate logistic regression analysis was conducted to determine which impulsivity dimensions were most strongly associated with BPD group membership, while controlling for clinical and demographic covariates, including gender, depressive symptoms (BDI-II score), and trait anxiety (STAI-Trait score).

As shown in Table 6, both Negative Urgency and Positive Urgency emerged as independent predictors of BPD diagnosis. Specifically, participants scoring above the cut-off of 11 on Negative Urgency were 5.31 times more likely to belong to the BPD group (adjusted OR = 5.31; 95% CI: 2.07–13.62; *p* = 0.001). Likewise, individuals with scores above 13 on Positive Urgency had a 3.26-fold higher likelihood of being classified in the BPD group (adjusted OR = 3.26; 95% CI: 1.37–7.75; *p* = 0.007).

These findings underscore the central role of emotional impulsivity, expressed both in negative and positive affective contexts, in the psychopathological profile of BPD. They further support the clinical relevance of these UPPS-P dimensions as differential markers and potential therapeutic targets.

### 3.4. Correlations Between Impulsivity Dimensions and Clinical Variables Within the BPD Group

In the BPD group, Kendall’s tau-b correlations indicated significant positive associations between depressive and anxiety symptoms (BDI-II and STAI-T scores) and several UPPS-P dimensions. Both depression and trait anxiety were correlated with Negative Urgency, Positive Urgency, Lack of Premeditation, and Lack of Perseverance, with the strongest associations observed for Lack of Perseverance (τ ≈ 0.30, *p* < 0.05). By contrast, no significant correlations were found between UPPS-P dimensions and the severity of borderline symptomatology as assessed by the DIB-R (total score or subscales) (see Table 7).

Regarding risk-related behaviors, Kendall’s tau-b analyses revealed that the number of suicide attempts was positively correlated with the overall severity of borderline symptomatology as measured by the DIB-R total score (τ ≈ 0.30, *p* < 0.05), as well as with depressive symptoms (BDI-II; τ ≈ 0.24, *p* < 0.05) and trait anxiety (STAI-T; τ ≈ 0.26, *p* < 0.05). In addition, substance use was significantly associated with the impulsivity subdimension of the DIB-R (τ ≈ 0.29, *p* < 0.05) and also showed a weaker but significant association with trait anxiety (STAI-T; τ ≈ 0.15, *p* < 0.05) (see Table 8).

## 4. Discussion

The primary objective of this study was to better characterize the impulsivity profile of young adults with BPD, using the multidimensional short UPPS-P model as a theoretical and measurement framework.

We formulated four main hypotheses: that individuals with BPD would exhibit significantly higher scores on specific UPPS-P dimensions—namely Negative Urgency, Positive Urgency, and Lack of Premeditation—compared to a control group; that Negative Urgency would emerge as a robust predictor of BPD diagnosis independently of depressive and anxiety symptoms; that the severity of borderline symptoms, as measured by the DIB-R, would correlate with certain impulsivity dimensions of the UPPS-P; and finally, that impulsivity traits would be associated with clinical variables commonly linked to emotional dysregulation, such as depression, trait anxiety, and substance use.

The analyses conducted provided partial yet significant support for these hypotheses while also revealing several clinical and methodological nuances.

In particular, our findings highlight the central role of emotional impulsivity, especially Negative Urgency, in the psychopathology of young adults with BPD, reinforcing the relevance of a dimensional and individualized understanding of the disorder.

These results must be interpreted in light of the developmental context specific to this age group. The period from 16 to 25 years old represents a critical stage of psychosocial development marked by neurobiological vulnerabilities—such as incomplete maturation of prefrontal regions involved in impulse control—alongside psychological challenges including identity formation and the quest for autonomy, as well as major social transitions such as school completion, entrance into higher education or the workforce, and the assumption of new responsibilities [50,51]. Such transitions may exacerbate emotional dysregulation and contribute to the emergence or consolidation of impulsive traits characteristic of BPD, especially Negative Urgency. Impulsivity must therefore be considered in close interaction with the developmental context in which it is expressed [52]. We acknowledge, however, that emerging adulthood is a heterogeneous stage, with considerable individual variability in the timing and intensity of these neurodevelopmental and psychosocial transitions. This variability may contribute to the heterogeneity of impulsivity profiles observed within this age group.

The demographic characteristics observed in our sample are broadly consistent with those reported in the literature on BPD. The striking female predominance in the BPD group (over 95%) reflects classic epidemiological findings that suggest a female-to-male diagnostic ratio of approximately three to one. However, this overrepresentation may not reflect a true difference in prevalence, but rather result from various biases, including a greater tendency among women to seek psychological help, higher clinical sensitivity to internalizing symptoms in women, and a relative under-recognition of externalizing manifestations of BPD in men, which may instead be diagnosed as conduct disorders or substance-related disorders [1,53].

Our study also confirms that young adults with BPD present higher rates of psychiatric comorbidities, greater use of psychological care, and significantly more frequent substance use (tobacco, cannabis, other illicit drugs) compared to the control group. These findings are consistent with previous studies emphasizing the particularly high burden of comorbidities among young individuals with BPD, relative to other psychiatric conditions. Such comorbidities—including mood disorders, anxiety, and ADHD—contribute to the clinical complexity of BPD and highlight the importance of multimodal and individualized therapeutic strategies [1,54].

In addition, more than 77% of participants with BPD reported a history of suicide attempts, a striking figure that is consistent with prior literature linking BPD to heightened impulsivity and emotional dysregulation. Such behaviors are frequently exacerbated during adolescence and early adulthood, periods marked by heightened emotional and identity instability [52,55].

Another relevant vulnerability factor identified in our sample relates to the significantly higher prevalence of familial psychiatric history among individuals with BPD. These findings support the hypothesis of intergenerational transmission of psychopathological vulnerability, particularly in cases where paternal substance use or maternal borderline traits are present. Such backgrounds may contribute to the early onset of difficulties in emotional regulation, attachment, and risk behaviors [2,45].

In contrast to the literature, no significant group difference was observed regarding somatic comorbidities. This may be due to the relatively young age of the sample (median age: 17), as somatic complications associated with BPD—often resulting from risky behaviors such as substance abuse, eating disorders, or self-injury—tend to emerge or intensify later in adulthood [56].

Psychometric data show that participants with BPD displayed significantly higher scores on both the BDI-II and the STAI-Trait anxiety scale compared to the control group. These findings align with the extensive literature describing BPD as a disorder with high emotional comorbidity, frequently associated with severe depressive and anxious symptomatology [52,57].

The average BDI-II score in the BPD group reached a level categorized as “severe,” indicating substantial subjective distress and emotional suffering. This result is clinically relevant given that depression in the context of BPD is often linked to a heightened risk of suicide [58]. Mood disturbances in these individuals tend to be reactive to interpersonal stressors, making their assessment and treatment more complex than those of classic unipolar depression [59].

Similarly, elevated STAI-T scores suggest a stable disposition toward experiencing anxiety across various situations, regardless of immediate context. This trait-like vulnerability appears to play a major role in amplifying emotional reactivity and impulsive behaviors [60].

Despite the inclusion of depression and anxiety scores as covariates in our multivariate analyses, emotional impulsivity—particularly Negative Urgency—remained significantly associated with BPD diagnosis This suggests that impulsivity in BPD cannot be attributed solely to the presence of comorbid depressive or anxious symptoms but rather constitutes an autonomous psychopathological trait intrinsic to the disorder. The simultaneous presence of high depressive and anxious symptom scores further supports the need for integrated psychotherapeutic approaches aimed at regulating emotional intensity, managing negative affect, and preventing behavioral crises. These results align with the theoretical and clinical rationale behind treatments such as Dialectical Behavior Therapy (DBT), which target impulsivity, emotional instability, and affective distress [1,10,15].

### 4.1. Negative Urgency: A Central Clinical Marker of BPD

Our findings support the centrality of impulsivity in BPD while emphasizing the emotional nature of this construct. Based on the UPPS-P model, our analyses show that not all facets of impulsivity contribute equally to the clinical differentiation of BPD: only Negative Urgency, and to a lesser extent Positive Urgency, significantly discriminate between BPD and control groups.

Among these, Negative Urgency emerges as the most powerful marker of impulsivity in young adults with BPD. This dimension reflects a tendency to act rashly under the influence of intense negative emotions such as anger, anxiety, shame, or psychological pain [22,23]. Our results indicate that Negative Urgency is not only elevated in the BPD group but also independently predicts BPD status, even after adjusting for depressive symptoms, anxiety, suicidality, and psychiatric comorbidities. Specifically, individuals scoring above the cut-off of 11 on Negative Urgency were more than five times more likely to be classified in the BPD group (adjusted OR = 5.31; 95% CI: 2.07–13.62; *p* = 0.001).

However, this finding should still be interpreted with caution. Although we met the standard methodological requirements for multivariate logistic regression—including an adequate subject-to-variable ratio (≥10 per cofactor)—and validated the model using robust statistical methods (robust standard errors, Hosmer–Lemeshow test, and Link test), the relatively small sample size may nonetheless lead to wide confidence intervals and reduced precision in effect estimation. Therefore, while our results support previous research identifying Negative Urgency as a relevant clinical marker of BPD [5,17], and align with explanatory frameworks such as Linehan’s biosocial theory (1993) [10], these findings should be considered preliminary and require replication in larger, adequately powered samples before drawing firm conclusions about its standalone predictive value.

Neurobiological evidence supports this view, with brain imaging studies in BPD patients showing disorganized fronto-limbic circuits, including amygdala hyperactivation and prefrontal hypoactivation in response to negative stimuli [17,21].

Negative Urgency thus does not reflect a global inhibition deficit but rather a specific vulnerability to emotionally triggered impulsive responses. This emotional and neurocognitive specificity suggests that Negative Urgency could be considered a key therapeutic target, particularly within interventions focused on emotional regulation such as DBT [10].

Moreover, the absence of significant effects for the other UPPS-P dimensions (Lack of Premeditation, Lack of Perseverance, and Sensation Seeking) does not necessarily imply that these traits are irrelevant to BPD. Given the modest sample size, the study was likely underpowered to detect small to moderate effects, increasing the risk of type II error. In this context, non-significant results may reflect insufficient statistical power rather than a true lack of association.

### 4.2. Positive Urgency in BPD: A Significant but Less Potent Predictor

Positive Urgency—defined as the tendency to act impulsively under the influence of intense positive emotions such as excitement or euphoria [22,23]—was also found to be significantly elevated among participants with BPD compared to controls. Importantly, in our multivariate analyses, Positive Urgency remained an independent predictor of BPD status after adjusting for confounding variables. Specifically, individuals scoring above the cut-off of 13 were more than three times more likely to be classified in the BPD group (adjusted OR = 3.26; 95% CI: 1.37–7.75; *p* = 0.007).

This result is in line with previous work showing that impulsive behaviors in BPD may be triggered not only by negative affect but also by states of heightened positive arousal [30]. At the same time, converging evidence indicates that Positive Urgency is not unique to BPD: studies such as Waite et al. [5] have shown comparable elevations in other clinical populations, particularly in bipolar disorder and substance use disorders.

Taken together, these findings suggest that Positive Urgency contributes meaningfully to the impulsivity profile of BPD. While it is less potent than Negative Urgency in differentiating BPD from controls, its significance underscores the importance of considering both negative and positive affective contexts when conceptualizing impulsivity in borderline psychopathology. Although Positive Urgency has been described in the literature as a potential transdiagnostic vulnerability factor, our study was not designed to test this hypothesis across diagnostic categories.

Nevertheless, these findings should be interpreted with caution. Given the limited sample size, the absence of additional significant effects beyond Positive Urgency may reflect insufficient power rather than a true lack of association (type II error). Larger studies are warranted to confirm the specific role of Positive Urgency relative to other impulsivity dimensions.

### 4.3. Cognitive Impulsivity: Present but Nonspecific Traits

A detailed analysis of the impulsivity profile of young adults with BPD using the UPPS-P model highlights a key distinction between emotional and cognitive facets of impulsivity. Although all UPPS-P subscales (except Sensation Seeking) were significantly elevated in the BPD group in univariate analyses, only emotional dimensions—particularly Negative Urgency and, to a lesser extent, Positive Urgency—remained significantly associated with the diagnosis in multivariate models.

Unlike emotional impulsivity, cognitive components such as Lack of Premeditation (difficulty anticipating the consequences of actions) and Lack of Perseverance (tendency to disengage from effortful or boring tasks) did not retain predictive value after controlling for confounding variables. Although these traits were generally elevated among BPD participants, they did not serve to specifically distinguish the disorder.

Although our results show that Lack of Premeditation was elevated among individuals with BPD, previous studies suggest that this trait is also present in other clinical populations such as those with ADHD, substance use, or mood disorders [14,43]. This supports the notion of its potential transdiagnostic relevance, which warrants further investigation in comparative studies.

Lack of Perseverance, a dimension still underexplored in the context of BPD, may reflect low tolerance for effortful cognitive processing under emotional constraint, difficulty maintaining attention, or frustration intolerance [35], but does not appear to be a core feature of the disorder. These null findings deserve to be discussed in light of studies highlighting the specificity of emotional impulsivity in borderline pathology, to the detriment of more cognitive components. Indeed, several recent studies have failed to establish a consistent relationship between executive function deficits and borderline symptoms, particularly in nonclinical populations. For instance, Veerapandian et al. [44], using a latent variable modeling approach in a sample of 233 young adults, found that neither inhibition, cognitive flexibility, nor working memory updating were significantly associated with borderline symptomatology. The authors suggest that, in nonclinical populations, borderline symptoms may be less rooted in stable cognitive dysfunctions, and more influenced by affective, reactive, or contextual factors [44].

This perspective reinforces the idea that affect-driven impulsivity traits, such as negative and positive urgency, may be more directly implicated in borderline mechanisms—particularly among individuals without a clinical diagnosis. It also helps to contextualize the lack of association observed here between borderline traits and “cold” impulsivity facets such as lack of premeditation, which aligns with a broader literature reporting inconsistent or null effects in this domain.

Sensation Seeking, for its part, showed no significant differences between groups in our sample. This finding is consistent with previous literature suggesting that this trait is not systematically elevated in BPD, particularly in controlled experimental conditions.

It is more commonly observed in externalizing disorders such as antisocial behavior or substance use and may be influenced by environmental or emotional context [5,28,39].

In summary, our findings suggest that the clinical specificity of BPD does not lie in global impulsivity but in a dysregulated, context-dependent form of emotional impulsivity centered on behavioral responses to intense negative affect. This distinction between emotional and cognitive impulsivity confirms the usefulness of the UPPS-P model in refining our understanding of the borderline psychopathological profile.

The lack of significant associations involving cognitive impulsivity traits (Lack of Premeditation, Lack of Perseverance) should also be considered in light of potential type II error. With the present sample size, small or moderate effects may not have been detected. Future research with larger samples is needed to determine whether cognitive impulsivity contributes meaningfully to BPD pathology.

### 4.4. Impulsivity: Between Stable Traits and Symptomatic Expressions

A particularly notable finding of this study is the absence of significant correlations between UPPS-P subscales and dimensional scores of the DIB-R, whether affective, cognitive, impulsive, or interpersonal. Two main hypotheses may account for this dissociation.

From a methodological standpoint, the lack of association may be due to the limited statistical power of the clinical subsample (*n* = 44), combined with the use of a strict Bonferroni correction to minimize type I error. These factors may have reduced the sensitivity needed to detect moderate effect sizes. From a conceptual standpoint, the dissociation highlights the distinct nature of the two instruments. The UPPS-P aims to assess stable personality traits of impulsivity, considered relatively consistent across time and context [23,24], while the DIB-R evaluates current clinical symptoms of BPD, which may fluctuate over time and be more sensitive to situational or interpersonal stressors [47]. This suggests that the lack of correlation does not necessarily reflect inconsistency but rather a complementary relationship between a dimensional trait-based assessment (UPPS-P) and a syndrome-based clinical tool (DIB-R). This interpretation is supported by broader findings in the literature showing that different methods used to assess impulsivity—self-reports, clinical observation, behavioral tasks—do not necessarily measure the same facets of the construct [19,32]. Although our study did not include neuropsychological tasks, the data suggest that a multimodal approach integrating subjective and clinical evaluations is essential to fully grasp the complexity of impulsivity in BPD, which spans both stable dispositional traits and fluctuating symptomatic expressions.

It is also worth noting that all participants in the BPD group were previously diagnosed by licensed psychiatrists, and the DIB-R was administered post hoc, not to determine inclusion, but to provide a dimensional evaluation of symptom severity. Consequently, the lack of correlation between DIB-R subscales and UPPS-P traits does not reflect a failure of diagnostic alignment but rather highlights a possible dissociation between clinical symptom severity and dispositional emotional impulsivity. While the DIB-R captures state-sensitive symptom clusters, the UPPS-P targets relatively stable personality traits, which may not covary linearly with current clinical expression. This interpretation aligns with previous findings on the divergence between trait-based and syndrome-based measures of impulsivity [19,32].

A statistical explanation worth considering lies in the potential restriction of variance on DIB-R scores within the BPD group. As all participants had a confirmed clinical diagnosis, symptom severity was generally high across the sample. This homogeneity in clinical presentation may have limited the variability necessary to detect meaningful correlations with trait-level impulsivity dimensions. In such cases, even theoretically relevant associations may be attenuated or suppressed due to insufficient distributional spread.

Another important consideration is the clinical heterogeneity of BPD presentations, which may have contributed to the lack of associations. BPD is a multifaceted disorder encompassing a wide range of symptom expressions, from internalizing features (e.g., affective instability, depressive states) to externalizing tendencies (e.g., impulsive aggression, substance use). Given this variability, it is possible that only certain subtypes within the BPD group exhibit strong links between specific UPPS-P traits and symptom dimensions. This unmodeled heterogeneity could have obscured associations in the aggregate analyses.

It is also important to emphasize that our statistical approach included a Bonferroni correction to adjust for multiple comparisons within the clinical subgroup. While this procedure is effective in controlling type I error rates, it is known to be highly conservative, particularly in studies with limited sample sizes. In the context of our BPD subsample (*n* = 44), this adjustment may have reduced the statistical power to detect medium-sized effects, thereby increasing the likelihood of type II errors. Consequently, some theoretically meaningful associations—such as those between DIB-R subscales and specific UPPS-P traits—may have failed to reach statistical significance despite underlying associations.

Beyond the absence of associations with DIB-R scores, our analyses revealed that depressive and anxiety symptoms (BDI-II and STAI-T) were significantly correlated with several UPPS-P dimensions, particularly Negative Urgency and Lack of Perseverance. This finding underscores the close interdependence between emotional dysregulation and dispositional impulsivity in BPD, suggesting that mood and anxiety symptoms may amplify the expression of impulsive tendencies [5].

From a clinical perspective, these results highlight the importance of a dual-level assessment strategy. On one hand, self-report measures such as the UPPS-P capture relatively stable dispositional traits of impulsivity. On the other hand, clinician-rated instruments such as the DIB-R provide a complementary evaluation of state-dependent symptom clusters and their behavioral consequences. Integrating both perspectives in clinical practice may improve the precision of diagnostic formulations and inform tailored therapeutic interventions—targeting not only acute symptom fluctuations but also the underlying dispositional traits that sustain vulnerability to emotional and behavioral dysregulation.

### 4.5. Risk Behaviors: Impulsivity, Substance Use, and Suicide Attempts

Our within-group correlation analyses in the BPD sample revealed clinically meaningful associations between certain risk behaviors and both symptom severity and emotional states. Specifically, the number of suicide attempts was positively correlated not only with the global severity of BPD symptoms (DIB-R total score), but also with depressive (BDI-II) and anxiety (STAI-T) symptoms. Similarly, substance use (cannabis and other illicit drugs) was associated with the impulsivity dimension of the DIB-R and also showed a significant correlation with trait anxiety. These findings suggest that self-damaging (suicidal) and addictive (substance-related) behaviors may reflect both the behavioral consequences of emotional impulsivity and the amplifying role of affective symptomatology, particularly in contexts of acute psychological distress.

They underscore the necessity of systematically integrating such clinical factors—symptom burden, impulsivity, and emotional dysregulation—into suicide risk assessments as well as into the planning of care pathways and therapeutic interventions [58,61].

Moreover, the absence of significant correlations between UPPS-P scores and DIB-R dimensions suggests that the type of emotional impulsivity captured by the UPPS-P may reflect a more stable dispositional style, relatively independent of momentary fluctuations in depressive or anxious symptoms. This supports its conceptualization as a transdiagnostic trait rather than a purely symptomatic state.

Altogether, these data support a dual-level conceptualization of impulsivity in BPD—as both a structural vulnerability factor [5] and a proximal trigger for behavioral acting-out in crisis situations, particularly involving suicidal or addictive behaviors [58,61]. Such a framework calls for integrated clinical interventions that target not only acute symptomatology but also underlying personality traits contributing to emotional and behavioral dysregulation.

### 4.6. Justification for the Inclusion of Clinical Variables: Depression, Trait Anxiety, and Substance Use

The inclusion of variables such as depression (assessed via the BDI-II), trait anxiety (measured using the STAI-T), and substance use (tobacco, alcohol, illicit drugs) was grounded in empirical evidence highlighting their relevance in the expression of impulsivity, including among young adults with BPD.

Although the literature focusing specifically on this age group remains limited, several studies suggest that these psychological dimensions act as affective and behavioral modulators, shaping the form, intensity, and frequency of impulsive behaviors in emotionally dysregulated contexts [35,58,62].

In the case of depression and trait anxiety, numerous studies have underscored their central role in amplifying emotional impulsivity, particularly in its Negative Urgency component.

This facet—characterized by rash action in response to intense negative emotions—is highly sensitive to psychological distress. Waite et al. [5] report that Negative Urgency is significantly heightened in affective suffering, particularly in relation to anxious or depressive affect. Similarly, Gratz et al. [36] emphasize the role of low distress tolerance in triggering impulsive behavior among BPD patients Cyders and Coskunpinar [32] further suggest that the often modest associations between self-reported impulsivity traits and actual impulsive behavior may be influenced by contextual emotional variables such as depression or anxiety, which shape the behavioral expression of impulsivity.

These data justify the integration of the BDI-II and STAI-T as covariates in the multivariate models to isolate the specific effects of impulsivity traits and neutralize the confounding impact of co-occurring emotional distress.

Substance use emerges as a central factor in the study of impulsivity in BPD, particularly in young adults, for whom this comorbidity is especially prevalent [1,2]

Far from representing a secondary comorbidity, substance use can be conceptualized both as a behavioral manifestation of heightened emotional impulsivity—especially in its Negative Urgency and Lack of Premeditation dimensions [27,35,62,63] and as an aggravating factor contributing to the overall clinical severity of the disorder. These impulsivity profiles may predispose individuals to engage in substance use in contexts of acute emotional distress, with substances serving as short-term emotion regulation strategies despite awareness of their long-term negative consequences [43,62]. This short-term relief mechanism, driven by poor distress tolerance, reinforces the emotional and behavioral instability typical of BPD.

Beyond their clinical relevance, the combined inclusion of depression, trait anxiety, and substance use in the analytical models enhances internal validity by controlling for the confounding influence of co-occurring emotional states or maladaptive coping strategies.

This methodological approach aligns with an integrative psychopathological perspective, particularly suited to young adults with BPD, in whom personality traits, affective comorbidities, and maladaptive coping mechanisms intersect in complex ways. Although the variables included are described as transdiagnostic in the literature, in this study they were analyzed specifically in their role as modulators of impulsivity during this key developmental stage.

### 4.7. Strengths and Limitations of the Study

This study presents several methodological and clinical strengths. It focuses on a critical developmental period—ages 16 to 25—characterized by ongoing prefrontal maturation, identity formation, and major socio-professional transitions. This stage, often described as emerging adulthood, has been identified in prior research as a period of heightened vulnerability for the emergence and consolidation of borderline traits. The use of the multidimensional UPPS-P model, which is widely validated in clinical psychopathology, provided a nuanced framework for exploring distinct components of impulsivity in BPD. Furthermore, the combination of multiple validated instruments (short UPPS-P, DIB-R, BDI-II, STAI-T) with rigorous statistical analyses, including multivariate regressions and within-group correlations adjusted for relevant covariates, strengthens the robustness of our findings. Although the sample size was limited by clinical recruitment constraints, particularly in a transitional age population, the use of adjusted and robust statistical models (e.g., quantile regressions) aimed to mitigate this limitation and enhance the reliability of the observed effects.

The combined use of validated psychometric instruments (short UPPS-P, DIB-R, BDI-II, STAI-T) and complementary statistical analyses—including adjusted multivariate models and within-group associations—supports the reliability of several findings. In particular, the quantile regression analyses yielded robust and consistent effects for Negative Urgency, which emerged as a clinically relevant and specific marker of BPD.

Logistic regression models were strengthened through the use of heteroskedasticity-robust standard errors, ensuring a more reliable estimation of odds ratios and confidence intervals. Bivariate associations were computed using Kendall’s tau-b, a non-parametric correlation measure well-suited for ordinal data and small or tied samples.

These methodological choices enhance the robustness of our results. Nevertheless, due to the modest sample size, confidence intervals remain wide and effect sizes should be interpreted with caution. Despite these limitations, the identification of Negative Urgency as a potential differential marker of BPD offers meaningful insights for clinical assessment and targeted interventions on emotional impulsivity.

Several limitations must nevertheless be acknowledged. The relatively small size of the clinical subsample (n = 44) inevitably reduced statistical power, particularly in multivariate models and correlation analyses. This limitation is compounded by the strong female predominance (95.4%), which restricts the generalizability of findings to men. Gender may modulate impulsivity, and future studies should validate these findings in more balanced samples. The composition of the control group, although carefully defined to exclude psychiatric vulnerability and substance use, also introduces potential limitations. While this approach maximized the specificity of observed differences, it reduced ecological validity, as many young adults without formal psychiatric diagnoses nevertheless present psychological or behavioral vulnerabilities. The fact that controls were largely recruited from academic settings may also have introduced a sociocultural bias, limiting the representativeness of the sample.

A key limitation of this study is the potential selection bias linked to the recruitment of BPD participants from both inpatient and outpatient services, without exclusion based on clinical stability or comorbidities. This may have introduced variability in symptom expression and impulsivity levels. However, this approach also offers ecological validity, as it captures the clinical heterogeneity typically seen in BPD populations. Future studies might benefit from stratifying samples by clinical state or controlling for acute symptom severity to isolate trait-related mechanisms more precisely.

The absence of a psychiatric comparison group constitutes another limitation. By relying exclusively on a non-clinical control group, the study cannot establish whether the impulsivity dimensions observed are specific to BPD or shared with other psychiatric disorders such as mood disorders or ADHD. Furthermore, impulsivity was assessed exclusively through self-report questionnaires, which are inherently vulnerable to subjective biases linked to self-perception or current emotional state. The absence of complementary behavioral or neurocognitive measures reduces the ecological validity of the data and precludes cross-validation with objective indicators.

ADHD, although clinically relevant in the context of impulsivity, could not be adequately considered as a confounding variable due to its very low prevalence in our sample (n = 5). While this small number makes it unlikely to have significantly biased the results, it nonetheless represents a limitation, as comorbid BPD–ADHD has been shown to be associated with higher impulsivity than either disorder alone. In addition, the cross-sectional design of the study does not allow for causal inference regarding the relationship between impulsivity and BPD symptom severity. Longitudinal studies will be needed to clarify developmental trajectories and the role of impulsivity in the persistence or progression of the disorder. Finally, the single-site recruitment at a university hospital in Brussels, together with strict inclusion criteria, further limits external validity and calls for caution when generalizing findings to other sociocultural contexts.

Altogether, these methodological strengths and limitations highlight both the value and the boundaries of the present study. While the findings support the central role of emotional impulsivity in BPD and provide valuable clinical insights, they must be interpreted with caution and confirmed by future studies with larger, more diverse, and longitudinal samples, incorporating multimodal assessments and clinical comparison groups.

### 4.8. Clinical Implications and Future Directions

The findings of this study offer several clinical implications for the assessment, conceptualization, and treatment of BPD in young adults.

The identification of both Negative Urgency and Positive Urgency as independent predictors of diagnosis suggests that emotional impulsivity—whether triggered by negative or positive affective states—plays a central role in the psychopathological dynamics of these individuals. Negative Urgency, reflecting the tendency to act rashly under intense negative affect, has been consistently associated with self-harm, substance use, and suicidal behavior [5,31,35,36]. Positive Urgency, in turn, highlights the risk of disinhibited behaviors arising in contexts of heightened positive arousal, such as excitement or euphoria. Therapeutic strategies aimed at addressing both dimensions of emotional impulsivity may therefore contribute to reducing behavioral disorganization and preventing high-risk behaviors in vulnerable patients.

From an assessment perspective, the use of dimensional models such as the UPPS-P [22,23,24] provides a valuable framework for understanding the heterogeneity of impulsivity in BPD. Our study confirms that certain subcomponents—especially Negative Urgency and Positive Urgency—are significantly elevated in this population. By identifying these specific dimensions, clinicians can tailor interventions to individual profiles, thus promoting more personalized and effective care. Therapeutic programs focused on emotional regulation, such as Dialectical Behavior Therapy (DBT) or Mentalization-Based Therapy (MBT), have demonstrated efficacy in reducing impulsive behaviors [15].

The findings also highlight the potential value of considering clinical variables that have been described in the literature as transdiagnostic factors, such as depressive symptoms, trait anxiety, and substance use. These variables may influence the intensity and frequency of impulsive behaviors, especially when interacting with certain personality traits. Although traditionally viewed as comorbidities, they appear to contribute, at least in part, to the variability in BPD symptom expression among young adults. Their inclusion in analytical models may therefore refine the interpretation of findings and support a more integrative understanding of psychopathology during this developmental stage.

Within this framework, impulsivity should not be regarded as an isolated symptom but as a transversal vulnerability factor whose regulation represents a meaningful therapeutic target.

From a research perspective, several avenues warrant further exploration. It would be particularly valuable to extend this type of study to clinical comparison groups such as individuals with ADHD, bipolar disorder, or substance use disorders, in order to clarify the specificity of the impulsivity profile associated with BPD [21,27,46]. The addition of objective behavioral measures and neurobiological data (e.g., functional imaging, fronto-limbic connectivity) could enhance the understanding of underlying mechanisms and link impulsivity more directly to clinical, developmental, and neurocognitive markers [17,39,42].

In sum, a multimodal and integrative approach to impulsivity—bridging dimensional models, developmental trajectories, and clinical variables commonly associated with multiple psychiatric conditions—appears essential for improving differential diagnosis, predicting clinical outcomes, and optimizing individualized therapeutic interventions for young adults with borderline traits.

These findings support a paradigm shift in which impulsivity is no longer viewed as a mere behavioral consequence of emotional distress but rather as a core therapeutic target at the intersection of emotion, cognition, and interpersonal environment.

## 5. Conclusions

In conclusion, this study provides an exploratory yet meaningful contribution to the understanding of impulsivity in young adults with BPD. Using the multidimensional UPPS-P framework, we found that emotional impulsivity—particularly Negative Urgency, and to a lesser extent Positive Urgency—differentiated BPD from controls, even after adjustment for depression, anxiety, and other clinical variables. Within the BPD group, depressive and anxiety symptoms were significantly correlated with several impulsivity dimensions, while risk behaviors such as suicide attempts and substance use were associated with both overall symptom severity and emotional states. These findings support a dual-level conceptualization of impulsivity in BPD, combining dispositional traits and state-dependent clinical manifestations.

Although limited by sample size and the absence of a psychiatric comparison group, the study underscores the importance of considering developmental stage and emotional comorbidities when assessing impulsivity in BPD. By focusing on emerging adulthood (16–25 years), a period of heightened vulnerability that remains underrepresented in the literature, and by applying robust statistical methods, we highlight the central role of emotional impulsivity in borderline psychopathology. Future research using larger, longitudinal, and multimodal designs is needed to confirm these preliminary findings and further clarify the dynamic interplay between impulsivity traits, affective symptoms, and risk behaviors.

## Figures and Tables

**Table 1:** Sociodemographic and General Clinical Characteristics of Participants (*n* = 160).

Variables	Categories	%	Controls (*n* = 116)	BPD Individuals (*n* = 44)	*p*-Value Chi^2^
Gender	Male (*n* = 34) Female (*n* = 126)	21.3% 78.7%	27.6% 72.4%	4.6% 95.4%	0.001
Student	No (*n* = 8) Yes (*n* = 152)	5.0% 95.0%	4.3% 95.7%	6.8% 93.2%	0.516
Somatic history	No (*n* = 118) Yes *(n* = 42)	73.8% 26.2%	76.7% 23.3%	65.9% 34.1%	0.165
Surgical history	No (*n* = 81) Yes (*n* = 79)	50.6% 49.4%	48.3% 51.7%	56.8% 43.2%	0.335
Psychiatric comorbidities	0 (*n* = 129) 1 (*n* = 16) ≥2 (*n* = 15)	80.6% 10.0% 9.4%	100.0% 0.0% 0.0%	29.6% 36.4% 34.0%	<0.001
Smoking	No (*n* = 133) Yes (*n* = 27)	83.1% 16.9%	90.5% 9.5%	63.6% 36.4%	<0.001
Alcohol	No (*n* = 48) Occasional (*n* = 94) Binge drinking (*n* = 18)	30.0% 58.8% 11.2%	34.5% 55.2% 10.3%	18.2% 68.2% 13.6%	0.132
Drugs	No (*n* = 139) Yes (*n* = 21)	86.9% 13.1%	100.0% 0.0%	52.3% 47.7%	<0.001
Family psychiatric history	No (*n* = 121) Yes (*n* = 39)	75.6% 24.4%	100.0% 0.0%	11.4% 88.6%	<0.001
History of suicide attempts	No (*n* = 126) Yes (*n* = 34)	78.8% 21.2%	100.0% 0.0%	22.7% 77.3%	<0.001
Psychological follow-up	No (*n* = 116) Ambulatory (*n* = 39) Hospital (*n* = 5)	72.5% 24.4% 3.1%	100.0% 0.0% 0.0%	0.0% 88.6% 11.4%	<0.001
Psychotropic treatment	No (*n* = 128) Yes (*n* = 32)	80.0% 20.0%	100.0% 0.0%	27.3% 72.7%	<0.001
Educational level	Primary education (*n* = 101) Secondary or higher education (*n* = 59)	63.1% 36.9%	60.3% 39.7%	70.5% 29.5%	0.237
	**Median** **(P25–P75)**				**Wilcoxon test**
Age (years)	18.0 (17.0–20.5)		18.0 (17.0–20.5)	17.0 (16.0–20.5)	0.051
Number of suicide attempts	0.0 (0.0–0.0)		0.0 (0.0–0.0)	2.0 (1.0–5.0)	<0.001

**Table 2:** Between-Group Comparison of Impulsivity Dimensions Assessed by the UPPS-P (*n* = 160).

Variables	Median (P25–P75)	Controls (*n* = 116)	BPD Individuals (*n* = 44)	Wilcoxon Test
UPPS urgencies negative	11.0 (8.0–13.0)	10.0 (8.0–12.0)	14.0 (11.0–15.0)	<0.001
UPPS urgencies positive	13.0 (10.0–14.0)	12.0 (10.0–14.0)	14.0 (12.0–16.0)	<0.001
UPPS lacks premeditation	9.0 (7.0–11.0)	8.0 (6.0–9.0)	11.0 (9.0–13.0)	<0.001
UPPS lacks perseverance	8.0 (6.0–10.0)	7.0 (5.0–9.0)	11.0 (9.0–12.0)	<0.001
UPPS seeks sensation	12.0 (9.0–14.0)	11.0 (9.0–14.0)	13.0 (9.0–15.0)	0.126

**Table 3:** Assessment of Depressive Symptoms and Trait Anxiety Using the BDI-II and STAI-T (*n* = 160).

Variables	Median (P25–P75)	Controls (*n* = 116)	BPD Individuals (*n* = 44)	Wilcoxon Test
BDI	14.0 (9.0–27.0)	12.0 (8.0–18.0)	33.0 (19.0–45.0)	<0.001
Speilberger–Trait	50.0 (39.0–61.0)	45.0 (36.0–51.0)	66.0 (57.0–72.0)	<0.001

**Table 4:** Scores on the Diagnostic Interview for Borderlines—Revised (DIB-R) Among Participants with BPD (*n* = 44).

Dimension	Median (P25–P75)
Affective Dimension	9.0 (8.0–10.0)
Cognitive Dimension	3.0 (2.0–4.0)
Impulsive Dimension	7.0 (5.0–8.0)
Interpersonal Dimension	10.0 (8.0–12.0)
DIB-R Total Score	9.0 (8.0–10.0)

**Table 5:** Adjusted results for UPPS (*n* = 160).

Variables	b_a1_ (ES) Controls vs. BPD Individuals
UPPS urgencies negative	9.9 (3.7) *
UPPS urgencies positive	4.8 (2.4) *
UPPS lacks premeditation	1.6 (3.0)
UPPS lacks perseverance	2.1 (2.7)
UPPS seeks sensation	3.1 (3.8)

ba1 (ES): quantile regression coefficient adjusted (standard error). These coefficients are the difference in median between Controls and BPD individuals, adjusted for gender, psychiatric comorbidities, smoking, drugs, family psychiatric history, number of history of suicide attempts, psychological follow-up, psychotropic treatment, BDI score and Spielberger–Trait score. * *p* < 0.05.

**Table 6:** Multivariate analyses (*n* = 160).

Variables	%	Controls	BPD Individuals	Model 1 OR Unadjusted (CI 95%)	*p*-Value	Model 2 OR Adjusted (CI 95%)	*p*-Value
UPPS urgencies negative ≤11 (*n* = 89) >11 (*n* = 71)	55.6% 44.4%	66.4% 33.6%	27.3% 72.7%	1 5.26 (2.44 to 11.37)	<0.001	1 5.31 (2.07 to 13.62)	0.001
UPPS urgencies positive ≤13 (*n* = 102) >13 (*n* = 58)	63.8% 36.2%	73.3% 26.7%	38.6% 61.4%	1 4.35 (2.09 to 9.09)	<0.001	1 3.26 (1.37 to 7.75)	0.007

Model 1 = Model unadjusted. Model 2 = Model adjusted for gender, BDI score and Spielberger–Trait score. UPPS urgencies negative. Adequacy of model 2: Hosmer–Lemeshow chi2 (*p* = 0.914). Specificity of model 2: Linktest (linear component *p* < 0.001 and nonlinear component *p* = 0.736). UPPS urgencies positive. Adequacy of model 2: Hosmer–Lemeshow chi2 (*p* = 0.862). Specificity of model 2: Linktest (linear component *p* < 0.001 and nonlinear component *p* = 0.945).

**Table 7:** Correlation BPD dimensions and UPPS dimensions (*n* = 44).

	DIB R Total	Affective Dimension	Cognitive Dimension	Impulsive Dimension	Interpersonal Dimension	BDI Score	Spielberger–Trait Score
UPPS urgencies negative	0.005	0.061	−0.046	0.098	−0.022	0.237 *	0.231 *
UPPS urgencies positive	0.094	0.154	0.056	0.050	0.089	0.233 *	0.213 *
UPPS lacks premeditation	−0.069	0.025	−0.152	0.143	−0.169	0.238 *	0.228 *
UPPS lacks perseverance	0.040	−0.143	−0.072	0.129	−0.022	0.305 *	0.304 *
UPPS seeks sensation	0.057	0.030	0.033	0.140	−0.061	0.050	0.051

* *p* < 0.05 after Bonferroni correction.

**Table 8:** Correlation BPD dimensions and other impulsive markers (*n* = 44).

	DIB R Total	Affective Dimension	Cognitive Dimension	Impulsive Dimension	Interpersonal Dimension	BDI Score	Spielberger–Trait Score
Number of suicide attempts	0.298 *	−0.024	0.129	0.179	0.140	0.237 *	0.263 *
Drugs consumption	−0.048	−0.164	−0.186	0.287 *	0.003	0.146 *	0.138 *

* *p* < 0.05 after Bonferroni correction.

## Data Availability

The corresponding author may provide the data for this study upon reasonable request.

## Appendix A

To better characterize the study population, we analyzed the presence of psychiatric comorbidities within the group of participants diagnosed with BPD. The frequencies and percentages of comorbid diagnoses are presented in Table A1. This table provides a detailed overview of the distribution of associated psychiatric disorders—including anxiety disorders, depressive disorders, autism spectrum disorder, ADHD, and other conditions—according to the primary diagnosis of BPD.

**Table A1:** Frequencies and percentages of comorbid psychiatric diagnoses in BPD group (*n* = 44).

DSM-5-TR Diagnosis	Total, *n* (%)
Major Depressive Disorder	10 (22.7%)
Generalized Anxiety Disorder	7 (15.9%)
Attention-Deficit/Hyperactivity Disorder	5 (11.3%)
Social Anxiety Disorder	4 (9.1%)
Obsessive–Compulsive Disorder	2 (4.5%)
Eating Disorders (Anorexia and Bulimia)	7 (15.9%)
Cannabis Use Disorder	3 (6.8%)
Substance Use Disorder (Other than Cannabis)	3 (6.8%)
Post Traumatic Disorder	6 (13.6%)
Bipolar Disorder	2 (4.5%)
Persistent Depressive Disorder (Dysthymia)	1 (2.3%)
Adjustment Disorder	1 (2.3%)
